# A study protocol of a randomized phase II trial of perioperative chemoimmunotherapy verses perioperative chemoimmunotherapy plus preoperative chemoradiation for locally advanced gastric (G) or gastroesophageal junction (GEJ) adenocarcinoma: the NeoRacing study

**DOI:** 10.1186/s12885-022-09786-9

**Published:** 2022-06-28

**Authors:** Menglong Zhou, Wang Yang, Yan Xuan, Wei Zou, Yaqi Wang, Zhiyuan Zhang, Jing Zhang, Miao Mo, Changming Zhou, Yuan Liu, Wenming Zhang, Zhaozhen Zhang, Yiping He, Weiwei Weng, Cong Tan, Lei Wang, Dan Huang, Weiqi Sheng, Huanhuan Li, Hui Zhu, Yan Wang, Lijun Shen, Hui Zhang, Juefeng Wan, Guichao Li, Hua Huang, Yanong Wang, Zhen Zhang, Xiaowen Liu, Fan Xia

**Affiliations:** 1 Department of Radiation Oncology, Fudan University Shanghai Cancer Center, 270 Dong‘an Road, Shanghai, 200032 People’s Republic of China; 2 Department of Oncology, Shanghai Medical College, Fudan University, Shanghai, 200032 People’s Republic of China; 3 Shanghai Key Laboratory of Radiation Oncology, Shanghai, 200032 People’s Republic of China; 4 Department of Cancer Prevention, Fudan University Shanghai Cancer Center, Shanghai, 200032 People’s Republic of China; 5 Department of Endoscopy, Fudan University Shanghai Cancer Center, Shanghai, 200032 People’s Republic of China; 6 Department of Pathology, Fudan University Shanghai Cancer Center, Shanghai, 200032 People’s Republic of China; 7 Department of Radiology, Fudan University Shanghai Cancer Center, Shanghai, 200032 People’s Republic of China; 8 Department of Gastric Surgery, Fudan University Shanghai Cancer Center, Shanghai, 200032 People’s Republic of China

**Keywords:** Locally advanced gastric cancer, Gastroesophageal junction adenocarcinoma, Preoperative chemoradiation, Immunotherapy, Gastrectomy

## Abstract

**Background:**

Perioperative chemotherapy (ChT) and preoperative chemoradiation (CRT) are both the standard treatments for locally advanced gastric cancer (LAGC). CRT can achieve a higher pathological complete regression (pCR) rate, but whether this higher pCR rate can be transformed into a long-term survival benefit remains inconclusive. Therefore, relevant studies are in progress. On the other hand, immunotherapy has been established for the first-line treatment of advanced gastric cancer (AGC) and has been widely explored in the perioperative setting. The combination of chemotherapy/radiotherapy and immunotherapy may have a synergistic effect, which will lead to a better antitumor effect. The preliminary reports of ongoing studies show promising results, including a further improved pCR rate. However, the preferred treatment combination for LAGC is still not established. To solve this problem, we are carrying out this randomized phase II trial, which aims to evaluate the efficacy and safety of perioperative chemotherapy plus the use of PD-1 antibody with or without preoperative chemoradiation for LAGC.

**Methods:**

Eligible patients with LAGC or gastroesophageal junction (GEJ) adenocarcinoma were randomized to receive perioperative ChT, PD-1 antibody, surgery with (Arm A) or without preoperative CRT (Arm B), and PD-1 antibody maintenance until one year after surgery. The primary endpoint of this study is that the pCR rate of Arm A will be significantly higher than that of Arm B. The secondary endpoints include the pathological partial regression (pPR) rate, R0 resection rate, objective response rate (ORR), event-free survival (EFS), overall survival (OS), safety and surgical complications. Moreover, several explorative endpoints will be evaluated to find and validate the predictive biomarkers of immunotherapy.

**Discussion:**

The results of the NeoRacing study will provide important information concerning the application of PD-1 antibody in LAGC patients during the perioperative setting. Meanwhile, the two treatment protocols will be compared in terms of efficacy and safety.

**Trial registration:**

ClinicalTrials.gov, NCT05161572. Registered 17 December 2021 - Retrospectively registered.

## Background

According to the recently updated GLOBOCAN 2020, gastric cancer (GC) ranks fifth and fourth in terms of the estimated number of new cases and the number of deaths worldwide, respectively [1]. Of note, the majority of the worldwide GC cases and deaths occur annually in China, accounting for 43.9% of the worldwide cases and 48.6% of the worldwide deaths [1]. In this large GC patient population in China, locally advanced gastric cancer (LAGC) accounts for approximately 70.8% of the cases [2]. Therefore, how to appropriately treat these patients and improve their survival is a serious challenge.

The main treatment of LAGC is surgery-based multidisciplinary therapy [3]. Perioperative ChT and preoperative CRT are both recommended for LAGC in the NCCN guidelines for GC. High-level evidence of preoperative CRT has been mainly found for GEJ tumors [4, 5], but there is relatively rare evidence for its use in middle and distal GCs. Therefore, perioperative ChT obtains a higher recommendation level than preoperative CRT for LAGC. Both of these therapies have been proven safe and effective [6–8]. Compared with perioperative ChT, preoperative CRT can achieve better short-term efficacy, such as by achieving a higher pCR rate and a decreased tumor stage [9]. However, whether this benefit can be transformed into a long-term survival benefit is unknown [10]. Several ongoing randomized controlled trials from the East and the West, such as the PREACT (NCT03013010) [11], Neo-CRAG (NCT01815853), TOPGEAR (NCT01924819) [12] and CRITICS-2 (NCT02931890) [13], are trying to provide the answer.

In 2012, a phase 2 trial (NCT02024217) was designed and conducted at our center, and this study aimed to evaluate the efficacy and safety of preoperative CRT for LAGC [8]. Due to the promising results observed in our phase 2 trial, a phase 3 randomized trial called PREACT (NCT03013010) was launched in 2017, which aimed to compare the efficacy of preoperative CRT with preoperative ChT for LAGC [11].

Major advances with immune checkpoint inhibitors (ICIs) have started to change the clinical practice (especially for the treatment and prognosis) for GC. With the publication of the results of the CheckMate-649 study and the ATTRACTION-4 study, ICIs have been successfully established as a first-line treatment for advanced gastric cancer (AGC). Clinical trials of ICIs combined with chemotherapy, radiotherapy and/or targeted therapies in the perioperative setting are being carried out. Available research data has shown that with the addition of ICIs in the neoadjuvant setting, the pCR rate can be substantially improved.

Tegafur gimeracil oteracil potassium capsule (S-1) plus oxaliplatin (SOX regimen) is the preferred ChT regimen in East Asian countries. The preliminary results of two large-scale randomized trials (RESOLVE [14], RESONANCE [15]) in China suggested that the neoadjuvant SOX regimen is beneficial in terms of the R0 resection rate, tumor shrinkage and survival prolongation. Patients in the neoadjuvant chemotherapy group achieved a longer DFS than patients in the control group. Moreover, the efficacy and safety of the SOX regimen plus CRT have been proven by our previous trials [8]. Therefore, in this study, we continued to use the SOX regimen. Sintilimab is a fully human IgG4 monoclonal antibody (mAb) that is registered as Tyvyt. It was developed by Innovent Biologics and Eli Lilly and Company [16]. In the Orient-16 trial (NCT03745170), sintilimab plus capecitabine/oxaliplatin significantly improved the OS and PFS in patients with gastric/GEJ adenocarcinoma, regardless of the PD-L1 expression. As sintilimab has a similar antitumor effect as nivolumab and pembrolizumab, has a better safety profile and has obvious economic advantages, we decided to use sintilimab in our study.

Positive peritoneal lavage cytology without visible gross peritoneal metastasis (CY1P0) is a special type of distant metastasis of GC, which is defined as stage IV disease in the American Joint Committee on Cancer (AJCC) guidelines for GC (8th edition) [17] and in the Japanese Classifications of Gastric Carcinoma [18]. It has been reported to be a key predictor of peritoneal dissemination [19] and poor prognosis [20–23]. Currently, the therapeutic strategies for GC with CY1P0 have not been fully defined [24]. Typically, palliative chemotherapy has been widely accepted as the standard therapy for stage IV GC patients globally [25, 26]. Moreover, curative-intent surgery was not indicated in GC patients with CY1 according to the treatment algorithm in the Japanese gastric cancer treatment guidelines [25]. However, radical surgery followed by postoperative chemotherapy is one of the most widely accepted therapeutic strategies for GC patients with CY1 in clinical practice and was established by a phase II trial called the CCOG0301 study [27, 28]. In addition, preoperative chemotherapy as an initial treatment for GC patients with CY1 is considered a promising treatment strategy, as systemic chemotherapy could eliminate the limited metastatic disease in some patients, could convert the metastatic disease to resectable disease and could achieve long-term survival after curative surgery [20, 29–31]. Therefore, there are currently no widely accepted treatment guidelines for CY1P0 GC patients.

Based on the above background, we designed this study, aiming to evaluate the safety and efficacy of perioperative SOX plus sintilimab with/without preoperative CRT. The study protocol of this trial, which has the acronym NeoRacing, is described in this article.

## Methods

### Study design

**Fig. 1:**
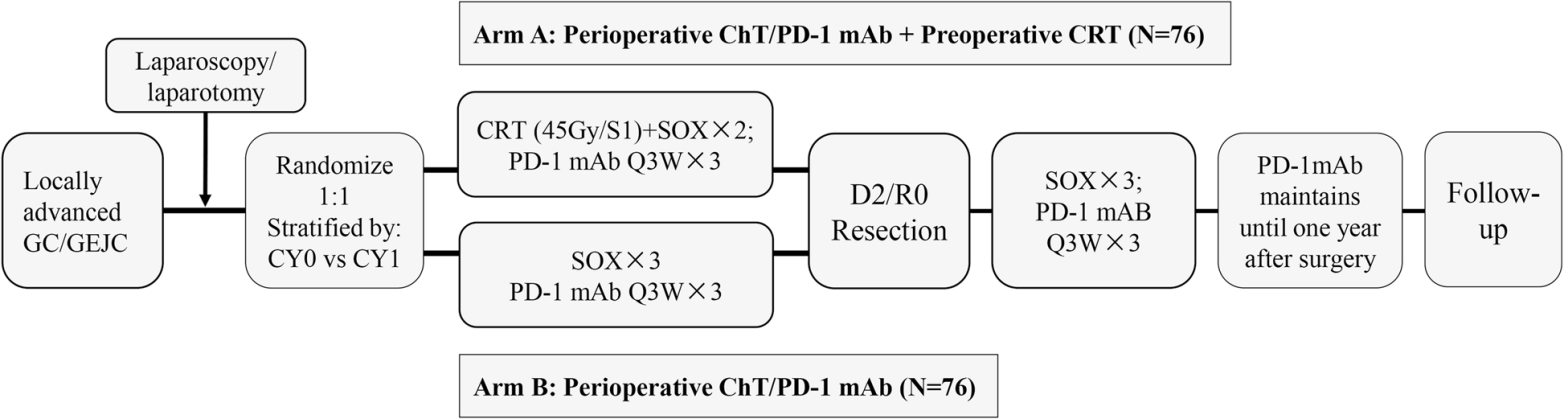
Flow chart of the NeoRacing study

NeoRacing is a randomized phase II trial carried out at Fudan University Shanghai Cancer Center (FUSCC) in China. The study can be divided into the screening stage, treatment stage and follow-up stage. The enrolled patients will receive perioperative SOX chemotherapy, PD-1 antibody (sintilimab) and radical surgery, with or without preoperative CRT (Fig. 1). The patients were randomized by stratified permutated block randomization on a web-based system. The status of peritoneal cytological examination (CY0 vs. CY1) was the stratification factor. The study protocol was approved by the Ethics Committee of FUSCC. All patients provided written informed consent before recruitment. Monitoring will be carried out in this trial.

### Primary endpoint

The primary endpoint is the pathological complete regression (pCR) rate: the proportion of patients who achieve pCR after preoperative therapy. Patients with a CY0 status at the time of enrollment should have no residual tumor cells in the primary lesion and the dissected lymph nodes in the surgical specimens (ypT0N0M0). Patients with a CY1 status at the time of enrollment should reach both ypT0N0M0 and a CY0 status.

### Secondary endpoints

1. The pathological response rate (pRR) is defined as the proportion of patients with pathological response. The tumor regression will be evaluated according to Ryan’s tumor regression grading (TRG) [32]. The pathological response is defined as TRG0 and TRG1 of the primary lesion after preoperative therapy.
2. The R0 resection rate is defined as the proportion of patients who achieve R0 resection. For patients with a CY0 status at the time of recruitment, the tumor should be completely removed, and no residual tumor cells within 1 mm of the resection margin should be confirmed by postoperative pathology. For patients with a CY1 status at the time of recruitment, an extra requirement is that CY0 should be confirmed by a peritoneal cytological examination.
3. The objective response rate (ORR) is defined as the proportion of patients with a complete response (CR) or a partial response (PR) to preoperative therapy. The ORR will be evaluated using the RESIST1.1 protocol.
4. Event-free survival (EFS)
5. Overall survival (OS)
6. Treatment safety
7. Surgical complications

### Explorative endpoints

1. The association of the baseline PD-L1 comprehensive positive score (CPS), tumor mutation burden (TMB), mismatch repair (MMR) status, and EBER with the efficacy of immunotherapy will be assessed.
2. The association between the gut microbiota and the efficacy of immunotherapy will be assessed.
3. The association between the *Helicobacter pylori* (HP) infection status and the efficacy of immunotherapy will be assessed.
4. The influence of the preoperative therapy on the tumor immune microenvironment (TIME) and the systemic immune status will be assessed.

### Inclusion criteria

1. Histopathologically confirmed gastric adenocarcinoma (G) or gastroesophageal junction adenocarcinoma (GEJ, excluding Siewert type I).
2. The clinical stage of the enrolled patients was cT3-4aN + M0 or cT4bNanyM0. Patients with a CY1 status but no other distant metastasis were allowed for patient recruitment. The clinical stage of CY1 patients is cT3-4aN + M1 (CY1 only) or cT4bNanyM1 (CY1 only) (the 8th AJCC staging system of GC).
3. The tumor was considered to be potentially resectable, which was verified by a multidisciplinary team including a surgical investigator.
4. At least one evaluable lesion on abdominal CT/MRI according to the RESIST 1.1 protocol is required.
5. An ECOG (Eastern Cooperative Oncology Group) performance status of 0-1.
6. The patient’s physical state and organ function can tolerate the planned treatment of the study protocol, including perioperative chemotherapy with the SOX regimen and immunotherapy with PD-1 monoclonal antibody, preoperative concurrent chemoradiotherapy (45 Gy/25 fractions/S-1), and major abdominal surgery.
7. The baseline laboratory examinations of the patients met the following criteria:
  - An adequate hematological function: an absolute neutrophil count (ANC) ≥ 1.5 × 10^9^/L; a platelet count ≥100 × 10^9^/L; a hemoglobin level ≥ 90 g/L.
  - Adequate liver function: total bilirubin ≤1.5 × upper limit of normal (ULN); AST/ALT < 2.5 × ULN; ALP ≤ 2.5 × ULN; ALB ≥30 g/L.
  - Adequate renal function: serum creatinine ≤1.5 × ULN; creatinine clearance rate ≥ 60 ml/min.
  - Adequate coagulation function: INR/PT ≤ 1.5 × ULN; APTT ≤1.5 × ULN.
8. There was no serious concomitant disease, and the patient’s life expectancy was more than 6 months.
9. Male or female. Age ≥ 18 years and ≤ 75 years.
10. Patients agreed to sign a written informed consent before recruitment.
11. Patients had good compliance with the study procedures, including laboratory examinations, auxiliary examinations and treatment.
12. The female patients should not be pregnant or breastfeeding.
13. The female patients agreed to take contraceptive measures during the treatment and within 120 days after the last dose of PD-1 mAb or 180 days after the last dose of chemotherapy or radiotherapy.

### Exclusion criteria

1. Clinical or histopathological evidence of peritoneal seeding (P1) or distant metastasis (M1).
2. Patients who have previously received surgery, chemotherapy, radiotherapy or immunotherapy for gastric cancer.
3. Patients had a history of cancer in the five years before randomization except for squamous or basal cell carcinoma of the skin that had been effectively treated and superficial bladder cancer, cervical carcinoma in situ and breast cancer in situ that had been treated by surgery.
4. Pregnant or lactating females or planning to become pregnant or lactating.
5. History of allergy to any drugs involved in this study.
6. History of allogeneic stem cell transplantation or organ transplantation.
7. Vaccinated with a live vaccine within 4 weeks before recruitment.
8. History of anti-PD-1, PD-L1, PD-L2 or any other specific T cell costimulation or checkpoint pathway targeted therapy.
9. History of using steroids (dose > 10 mg/d prednisone) or other systemic immunosuppressive therapy within 14 days before recruitment, except for patients treated with the following regimen: steroids used for hormone replacement (dose > 10 mg/d prednisone); local application of steroids with little systemic absorption; short-term (≤ 7 days) use of steroids to prevent allergy or vomiting.
10. Patients with weight loss of more than 20% within 2 months before recruitment.
11. Uncontrolled systemic diseases, including diabetes and hypertension.
12. Failure of important organs (heart, lung, liver, kidney, etc.).
13. Moderate or severe renal injury [creatinine clearance ≤50 ml/min (according to Cockroft & Gault equation)] or SCR > ULN.
14. Dipyrimidine dehydrogenase (DPD) deficiency.
15. Patients with central nervous system (CNS) disorders, peripheral nervous system disorders or psychiatric diseases.
16. A cerebrovascular accident that occurred within 6 months before recruitment.
17. Patients with a known history of uncontrolled or symptomatic angina, uncontrolled arrhythmias and hypertension, congestive heart failure, cardiac infarction within 6 months prior to study recruitment, or cardiac insufficiency.
18. Patients who have the following history of pulmonary diseases: interstitial lung disease, noninfectious pneumonia, pulmonary fibrosis, acute lung disease, or pulmonary embolism.
19. Patients with severe gastrointestinal bleeding, gastrointestinal perforation, or gastrointestinal fistula and patients who cannot swallow to take the drug orally.
20. Patients with upper gastrointestinal obstruction, dysfunction or malabsorption syndrome that can affect the absorption of oral chemotherapy drugs.
21. Uncontrollable pleural effusion, pericardial effusion, or ascites that occurred within two weeks before recruitment.
22. Patients with a history of active autoimmune disease or refractory autoimmune disease.
23. Severe chronic or active infections requiring systemic antibiotics, antifungal or antiviral therapy, including tuberculosis and AIDS.
24. Known history of human immunodeficiency virus (HIV) infection.
25. Patients with untreated chronic hepatitis B or HBV-DNA exceeding 500 IU/ml or HCV-RNA positivity.

### Treatment arms

During the screening stage, patients with LAGC or GEJ adenocarcinoma will undergo baseline examinations, including enhanced CT scans of the stomach, pelvic cavity and chest (if necessary). After preliminary screening, patients who may meet the inclusion criteria will receive laparoscopic exploration or laparotomy, and all of them will receive abdominal exfoliative cytology concurrently. Patients without gross peritoneal seeding (P0) will be finally enrolled. Eligible patients were randomized to two arms: the arm of perioperative ChT and ICI with preoperative CRT (Arm A) and the arm of perioperative ChT and ICI (Arm B).

### Treatments

#### Chemotherapy

**Fig. 2:**
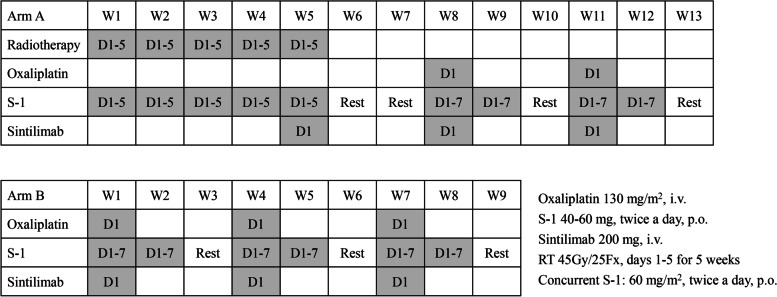
The treatment schedule of Arm A and Arm B in the NeoRacing study

The SOX regimen consists of S-1 and oxaliplatin and is repeated every three weeks. Oxaliplatin will be administered intravenously at 130 mg/m^2^ on day 1. S-1 is administered orally at 40-60 mg twice a day for 14 consecutive days. Then, the patients will have a one-week rest period (Fig. 2). The dose of S-1 is according to the body surface area (BSA): patients with a BSA of less than 1.25 m^2^ receive 80 mg daily; those with a BSA of 1.25 m^2^ or more but less than 1.5 m^2^ receive 100 mg daily; and those with a BSA of 1.5 m^2^ or more receive 120 mg daily.

#### Chemoradiation

In Arm A, CRT will be given upfront. The radiotherapy (RT) consists of 45 Gy delivered in 25 fractions every five days per week for five weeks. The dose of concurrent ChT is 60 mg/m^2^ S-1 orally, oral tablets twice daily, on days 1-5 of each week (Fig. 2). RT planning is performed using computed tomography (CT) images with intensity-modulated radiotherapy (IMRT) or volumetric modulated arc therapy (VMAT). The RT field includes the primary lesion, involved regional lymph nodes, as well as the draining lymph nodes areas. Four-dimensional CT (4DCT) or passive breath gating (PBG) is adopted to eliminate the influence of the respiratory movement.

#### Surgery

Gastrectomy with standard D2 lymphadenectomy is recommended. The type of gastrectomy performed depends on the location and extent of the primary lesion. For GEJ or upper third tumors, a 3 cm esophageal margin is recommended, and a total gastrectomy or esophagogastrectomy is performed. For middle third tumors, the gastric margin is recommended to be more than 5 cm, and a total gastrectomy is performed. For lower third tumors, a 2 cm duodenal margin is recommended, and a subtotal or total gastrectomy is performed. Billroth I or Roux-en-Y gastrojejunostomy is performed for distal gastrectomy patients. Roux-en-Y esophagojejunostomy is performed for patients receiving total gastrectomy.

#### Immunotherapy

The immunotherapy includes perioperative treatment and postoperative maintenance treatment. The PD-1 mAb used in the study will be sintilimab, and the dose is 200 mg intravenously every three weeks. Regardless of which arm, the courses of immunotherapy that will be received by the enrolled patients are the same: 3 times preoperatively, 3 times postoperatively, and then maintained until one year after surgery.

### Efficacy, safety and toxicity evaluation

The efficacy evaluations include radiological and pathological evaluations. The radiological evaluation is based on an enhanced CT scan of the stomach. In Arm A, the tumor response evaluations are taken after CRT and when the preoperative treatment is completed. In Arm B, the tumor response evaluations are taken when the preoperative treatment is completed. All these evaluations are performed according to the Response Evaluation Criteria for Solid Tumors (RECIST) 1.1. The pathological evaluation is mainly used to evaluate the degree of tumor regression on the gross surgical specimens by judging the tumor regression grading (TRG) based on Ryan’s grading system [32].

Adverse events were assessed according to the Common Terminology Criteria for Adverse Event (CTCAE) v5.0. The evaluation of surgical complications is mainly based on the Clavien-Dindo classification.

### Follow-up

The follow-up of all patients will be carried out according to the protocol: every three months for at least two years, every six months for the third to the fifth year after surgery, and then every 12 months for life. The follow-up contents include the physical examination, tumor marker examination, and radiological examination. Ultrasounds and CT scans will be performed alternately every 6 months. Gastric endoscopy will be performed once a year.

### Sample size calculation

The preliminary analysis of the PREACT study (NCT03013010) showed that preoperative chemotherapy with three cycles of the SOX regimen can yield a pCR rate of 2%, and two cycles of the SOX regimen plus chemoradiation of 45 Gy/S-1 yielded a pCR rate of 17%. The preliminary analysis of a study called NeoPLANET (NCT03631615) showed that the pCR rate of CapeOX plus camrelizumab combined with chemoradiation was 26.7% (4/15). According to poster 4036 of 2021 ASCO, four cycles of mFOLFOX6 plus camrelizumab yielded a pCR rate of 10% (5/52). Thus, with the addition of PD-1 mAb, the increased range of the pCR in both the preoperative ChT group and CRT group was approximately 8 to 10%, which is a reasonable range. Based on these research data, in this study, the pCR rate of Arm A is set as P1 = 26%, and the pCR rate of Arm B is set as P2 = 10%. The null hypothesis (H0): the pCR rate of Arm A is not better than that of Arm B, that is, P1-P2 ≤ 0. The alternative hypothesis (H1): the pCR rate of Arm A can be increased by 16% compared with Arm B, that is, P1-P2 = 16%.

The patients were randomly assigned to two arms at a ratio of 1:1. One side test is adopted, and if the error rate of type I (α) is set to 0.05, then 138 patients need to be enrolled, i.e., 69 in each arm, which can ensure that the test efficiency (1-β) reaches 80%. When the dropout rate is set to 10%, 152 patients are needed, with 76 in each group.

### Statistics

The EFS will be calculated from the date of randomization to the date of any event or censoring. The event is defined as below: (1) locoregional recurrence; (2) peritoneal seeding; (3) distant metastasis; (4) death of any reason; (5) tumor progression according to RESIST 1.1. The OS will be calculated from the date of randomization to the date of death or the date of the last follow-up. Survival will be estimated by using the Kaplan-Meier method, and differences between the survival curves will be examined with the log-rank test. Fisher’s exact test will be used to compare the patient characteristics between the two arms. Endpoints related to the rate, including the R0 resection rate, pathological response rate, toxicity occurrence, and surgical complications, will be compared between the two arms using the chi-square test. All statistical tests are two-sided. The level of significance is *P* < 0.05.

## Discussion

Before immunotherapy was widely used, perioperative ChT and preoperative CRT have been the standard treatments for LAGC. If stratified by tumor location, the evidence for the use of preoperative CRT is mainly for GEJ tumors, and the evidence for the use of preoperative CRT for middle or distal GC is relatively lacking. Compared with perioperative ChT, perioperative ChT plus preoperative CRT can improve the tumor withdrawal and decline, but whether this benefit can be transformed into a long-term survival benefit is still uncertain. Studies aiming to answer this question from both East and West are ongoing. With the establishment of immunotherapy in the first-line treatment of AGC, an increasing number of studies, including phase III trials, have explored the value of immunotherapy in the perioperative setting. The regimens include different combinations of ChT, RT, targeted therapy and immunotherapy. Therefore, at present, comparing the advantages and disadvantages of preoperative ChT and preoperative CRT seems to be outdated. Based on this background, we designed the NeoRacing study to evaluate the safety and efficacy of perioperative ChT plus PD-1 mAb with or without preoperative CRT, aiming to address which one is the superior treatment in patients with LAGC.

In the PREACT study, our center has accumulated some experience in the preoperative treatment of LAGC. Either three cycles of the SOX regimen or two cycles of the SOX regimen combined with CRT can be well tolerated by patients. The PREACT study requires that each enrolled patient undergo abdominal exploration with peritoneal cytological examination to obtain an accurate preoperative staging. Data have proved that this design is very necessary. The preliminary analysis showed that approximately one-third of the potentially eligible patients were P1 or CY1. Thus, this design is reserved in the NeoRacing study. However, unlike the PREACT study, P0CY1 patients were eligible for the NeoRacing study.

As mentioned before, a series of perioperative immunotherapy studies are underway, mainly single-arm studies in phase I-II, including single ICI, dual ICIs, ChT plus ICI, ChT plus RT plus ICI, or ChT plus antiangiogenic agents plus ICI. The endpoint of most studies is the pCR rate. According to the preliminary results of some studies, the pCR rate of a single ICI is only 3.2% [33]. Compared with ChT alone, ChT combined with ICI can improve the pCR rate. In poster 4036 from the 2021 ASCO (NCT03939962), mFOLFOX6 combined with camrelizumab yielded a pCR rate of 10% [33]. In poster 216 from the 2021 ASCO-GI (NCT04341857), FLOT combined with sintilimab yielded a pCR rate of 18.8% [34]. The combination of ChT, RT and ICI can obtain an even higher pCR rate. In the NeoPlanet study (NCT03631615), a combination of CapeOX and CRT was used for proximal GC and yielded a pCR rate of 26.7%, which has been updated to 33.3% in the 2021 ESMO. Another study named SHARED obtained a pCR rate of up to 42.1% [35]. However, the sample size of these studies is small, so more clinical data are needed.

Several explorative endpoints are being determined in the NeoRacing study to find predictive biomarkers of immunotherapy. The main obstacle to immunotherapy is that the response rate is not ideal. Therefore, on the one hand, we need to find predictive biomarkers of immunotherapy. PD-L1 CPS, MMR, TMB, and EBER are biomarkers that have been studied in GC, but there are still disputes. In this study, the predictive efficiency of these biomarkers will be further validated. Some studies have also shown that the gut microbiota [36] and HP infection [37] will have an impact on the efficacy of immunotherapy. On the other hand, to overcome the resistance to immunotherapy, including primary and secondary resistance, we hope to evaluate the changes in the tumor microenvironment and systemic immune status before and after preoperative treatment and to find the causes and mechanisms of treatment resistance.

Overall, the available study results suggest that the tumor regression obtained by preoperative immunotherapy alone is not ideal, and using a combination with traditional treatment methods such as ChT and/or RT is the way to obtain a good efficacy. Although the pCR rate has been further improved, we still need to answer the question of whether this benefit can be transformed into a survival benefit. To the best of our knowledge, the NeoRacing study is the first randomized controlled study to compare the safety and efficacy of perioperative ChT combined with immunotherapy with or without preoperative CRT.

## Data Availability

Not applicable in this study protocol. The data (such as efficacy and toxicity) produced during and after the trial are available from the corresponding author upon reasonable request.

## Abbreviations

AGC: Advanced gastric cancer
BSA: Body surface area
ChT: Chemotherapy
CPS: Comprehensive positive score
CRT: Chemoradiation
CTCAE: Common Terminology Criteria for Adverse Event
FUSCC: Fudan University Shanghai Cancer Center
GC: Gastric cancer
GEJ: Gastroesophageal junction
ICI: Immune checkpoint inhibitor
LAGC: Locally advanced gastric cancer
mAb: Monoclonal antibody
MMR: Mismatch repair
ORR: Objective response rate
OS: Overall survival
pCR: Pathological complete regression
PFS: Progression-free survival
pRR: Pathological response rate
RECIST: Response Evaluation Criteria for Solid Tumors
SOX: S-1 and oxaliplatin
TIME: Tumor immune microenvironment
TMB: Tumor mutation burden
TRG: Tumor regression grading
